# Possible mechanism of polyspermy block in human oocytes observed by time-lapse cinematography

**DOI:** 10.1007/s10815-012-9815-x

**Published:** 2012-06-14

**Authors:** Yasuyuki Mio, Kyoko Iwata, Keitaro Yumoto, Yoshiteru Kai, Haruka C. Sargant, Chizuru Mizoguchi, Minako Ueda, Yuka Tsuchie, Akifumi Imajo, Yumiko Iba, Kyoko Nishikori

**Affiliations:** Reproductive Centre, Mio Fertility Clinic, 2-1-1, Kuzumo-Minami, Yonago, 683-0008 Japan

**Keywords:** Polyspermy block, Time-lapse cinematography (TLC), Human fertilization process, Zona pellucida, Embryonic development

## Abstract

**Purpose:**

To analyze the fertilization process related to polyspermy block in human oocytes using an in vitro culturing system for time-lapse cinematography.

**Methods:**

We had 122 oocytes donated for this study from couples that provided informed consent. We recorded human oocytes at 2,000 to 2,800 frames every 10 s during the fertilization process and thereafter every 2 min using a new in vitro culture system originally developed by the authors for time-lapse cinematography. We displayed 30 frames per second for analysis of the polyspermy block during fertilization.

**Results:**

Three oocytes showed the leading and following sperm within the zona pellucida in the same microscopic field. The dynamic images obtained during the fertilization process using this new system revealed that once a leading sperm penetrated the zona pellucida and attached to the oocyte membrane, a following sperm was arrested from further penetration into the zona pellucida within 10 s.

**Conclusions:**

The present results strongly suggest the existence of a novel mechanism of polyspermy block that takes place at the zona pellucida immediately after fertilization. These findings are clearly different from previous mechanisms describing polyspermy block as the oocyte membrane block to sperm penetration and the zona reaction. The finding presented herein thus represents a novel discovery about the highly complicated polyspermy block mechanism occurring in human oocytes.

**Electronic supplementary material:**

The online version of this article (doi:10.1007/s10815-012-9815-x) contains supplementary material, which is available to authorized users.

## Introduction

The development of assisted reproductive technology (ART) has recently enabled the direct observation of human oocytes, revealing various mysterious phenomena involving the beginning of life. However, it is undeniable that frequent microscopic examinations of human early embryos may have negative effects on them, making it difficult to obtain reliable detailed information of human embryonic development from still images. We therefore developed an in vitro culture system for time-lapse cinematography (TLC), based on Payne et al. [13], to analyze the morphologically dynamic events occurring during early human embryonic development. This system enables non-invasive and continuous imaging of human oocyte fertilization and embryonic development.

Our previous dynamic analyses of the fertilization process in human oocytes and of human embryonic development using the in vitro TLC system [12] confirmed for the first time the detailed time course of sequential events during embryonic development and revealed novel phenomena [fertilization cone, cytoplasmic strand, and splitting of the inner cell mass (ICM)] under culture conditions in vitro [Supplementary Movie 1 (Online resource 1)]*.* Our further observation of the movie revealed another novel phenomenon that is likely to be involved in the mechanism of polyspermy block, wherein once the leading sperm has penetrated the zona pellucida (ZP) and attached to the oocyte membrane, any following sperm within the ZP stop penetrating immediately. To date, two types of polyspermy block were thought to exist in marine animals and mammals, including human (“oocyte membrane block” and “zona reaction”). In addition, the “oocyte membrane block” occurs in seconds, while an oocyte membrane depolarization block has not been detected in studies of mammalian oocytes [9]. We therefore consider that the phenomenon described herein differs from previously proposed mechanisms for polyspermy block [3,4], prompting us to re-analyze all of our TLC data covering the fertilization process. Herein, we provide the results of this second detailed TLC analysis, which we believe confirms the existence of a novel mechanism for the prevention of polyspermy.

## Materials and methods

**Fig. 1:**
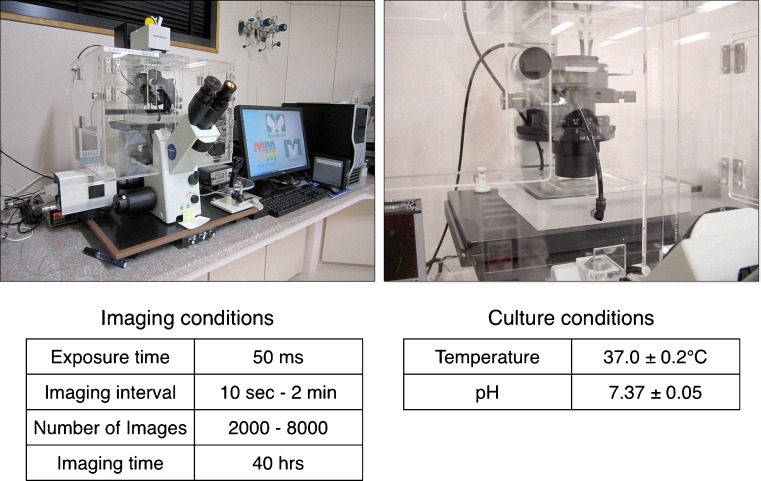
Time-lapse cinematography (TLC). The upper two images are from the TLC system, and the lower two tables indicate imaging and culture conditions of the TLC

The in vitro culture system for TLC has been described in detail elsewhere [12]. In brief, we used an inverted microscope (IX-71; Olympus, Tokyo, Japan) with Nomarski differential interference contrast optics (Olympus) and a micromanipulator (Narishige, Japan), which was covered by a handcrafted acrylic chamber. Our system also contained a small acrylic chamber surrounded by a small waterbath on the stage of the microscope, into which a glass Petri dish containing a microdrop of culture medium (5 μl) was placed. Humidified CO_2_ gas was infused into the chamber through the waterbath. The volume of flowing CO_2_ and temperature within the chamber were adjusted to give the optimal values (temperature, 37.0 ± 0.3 °C; pH, 7.37 ± 0.03). The inverted microscope was equipped with a CCD digital camera (Roper Scientific Photometrics, Tucson, AZ) connected to the computer and display by MetaMorph (Universal Imaging Co, Downingtown, PA) (Fig. 1).

Digital images of the cultured embryos were acquired for approximately 40 h by using an exposure time of 50 μs. In total, approximately 2,000–2,800 frames were taken during the observation period. We displayed movies at 30 frames per second to analyze the fertilization process and elucidate the mechanism of polyspermy block.

Before the commencement of TLC observation, we mechanically and gently removed the cumulus cells from around the oocytes, so as not to damage the tails of the sperm that penetrated into the ZP, at 1 h after the in vitro insemination (approximately 50 × 10^3^ sperm per oocyte). Images were acquired at 10-s intervals for the first 2 h, and thereafter at 2-min intervals, based on our preliminary study that showed most of the sperm penetrating the ZP within 3 h after the in vitro insemination.

Oocytes were collected from 122 couples after receiving informed consent, from July 2004 to December 2011. One oocyte was randomly selected from each couple and 122 oocytes were tested. In the TLC images obtained from these oocytes, penetration of the leading sperm into the ZP and attachment to the oocyte membrane was confirmed in the 22 oocytes. Among these, only three oocytes showed both the leading and following sperms within the ZP in the same frame, and these TLC data were therefore used to evaluate the dynamic changes in both sperms until the leading sperm attached to the oocyte membrane during the fertilization process.

The ethics committee of the Japanese Institution for Standardizing ART (JISART) approved our study protocol.

## Results

Of the 22 imaged oocytes, in which penetration into the ZP and attachment to the oocyte membrane of the leading sperm were confirmed, the leading sperm attached to the oocyte membrane within an average of 96 min after insemination, and the sperm head disappeared an average of 37 min after attachment of the sperm to the oocyte membrane. There was no difference in the time course of the fertilization process between the three oocytes subsequently chosen to analyze the mechanism for prevention of polyspermy and the remaining 19 oocytes.

**Fig. 2:**
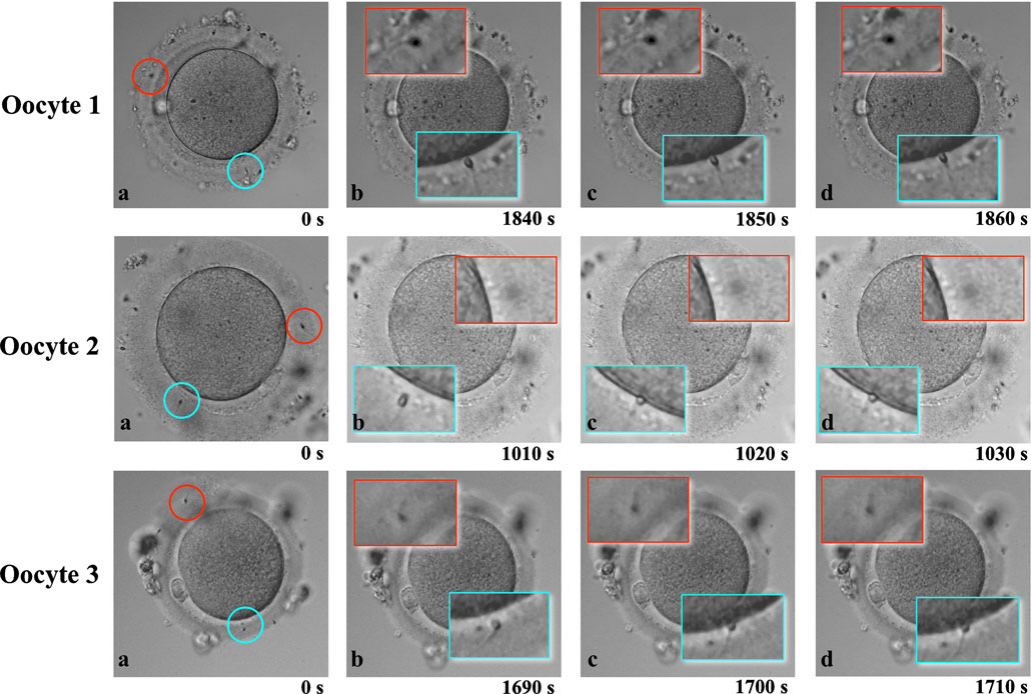
Dynamics of the leading sperm and following sperm. The sperm fertilizing the oocyte (leading sperm) is indicated by a *blue circle*, while the sperm following the fertilizing sperm (following sperm) is indicated by a *red circle* among three oocytes (**a**). Sections of the images containing both leading and following sperms were magnified to facilitate observation of the process (**b**, **c**, **d**). The images were acquired in 10-s intervals. With regard to oocyte 1 shown in the upper panel, both the leading and following sperm penetrated into the zona pellucida from the beginning of imaging to shortly prior to the attachment of the leading sperm to the oocyte membrane (**a**, **b**). The leading sperm attached to the oocyte membrane 1,850 s (30.8 min) after the beginning of imaging (**c**), and the penetration of the following sperm was inhibited at 1,860 s (**d**), which is within 10 s after the attachment of the leading sperm to the oocyte membrane. With regard to oocyte 2 shown in the middle panel and oocyte 3 shown in the lower panel, the penetration of the following sperm was also inhibited within 10 s after the attachment of the leading sperm to the oocyte membrane (**c**, **d**)

Figure 2 shows the results of analyzing each TLC frame of the selected oocytes (Oocyte 1, 2 and 3), in which the following sperm penetrated the ZP together with the leading sperm. Penetration of the following sperm into the ZP was arrested within 10 s after the leading sperm attached to the oocyte membrane, even though the tail of the following sperm was still actively moving in all three oocytes (Fig. 2). Additional data are given in Supplementary Movies 2 to 4 (Online Resource 2 to 4).

**Fig. 3:**
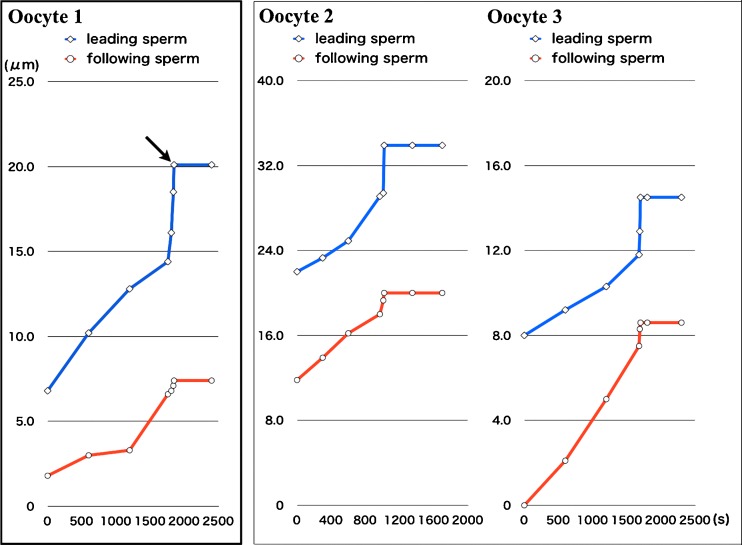
Change in the distance between the surface of the zona pellucida and leading or following sperm. The time course of the change in zona pellucida penetration distance of the leading sperm (*blue line*) and the following sperm (*red line*) shows that the zona pellucida penetration of the following sperm was inhibited when the leading sperm attached to the oocyte membrane (indicated by the *arrow*) after zona pellucida penetration in all oocytes (Oocyte 1, 2 and 3) used in this analysis

Next, we analyzed the time course of the distance that the leading and following sperms traveled in the ZP (Fig. 3). The following sperm traveled at a similar velocity to the leading sperm, until the leading sperm attached to the oocyte membrane after penetrating the ZP. However, once the leading sperm reached the oocyte membrane across the perivitelline space, the following sperm immediately ceased further penetration, within 10 s. The behaviors of the leading and following sperm were identical among the three oocytes.

## Discussion

The present work analyzed TLC imaging of the fertilization process in human oocytes. We revealed a novel phenomenon that is likely to be associated with the polyspermy block in human oocytes. This new mechanism involved cessation of the following sperm penetrating the ZP within 10 s of the leading sperm penetrating the ZP and attaching to the oocyte membrane. This was despite the following sperm retaining active tail movement and both sperm traveling at a similar velocity within the ZP before penetration. The behaviors of the leading and following sperm were identical among the three oocytes imaged and analyzed. The difficulty in obtaining TLC images in which both the leading and following sperm within the ZP were clearly identified precluded analysis of their respective behaviors in more than three oocytes.

Achieving normal fertilization of a single egg by a single sperm is core to life in many sexually reproducing animals, including humans. In most mammalian reproduction processes, huge numbers of sperm are ejaculated into the female reproductive tract (ratio of sperm to oocyte is approximately 10^9^:1); however, the vast majority of sperm are rapidly eliminated from the female tract [15]. It is thought that polyspermy is usually prevented by a decreased number of sperm reaching the Fallopian tube and a block mechanism in the fertilized oocyte [4].

In this context, it is also thought that in vitro fertilization (IVF) involves different mechanisms from the normal in vivo process in mammals. In particular, successful fertilization in vitro of a single oocyte requires a large number of motile sperms, and thus there is always the possibility that more than one sperm could penetrate the ZP and reach the oocyte [10]. However, between a healthy mature ovum and a healthy sperm, normal fertilization of a single egg by a single sperm occurs at a relatively high rate, which can also be confirmed easily in the environment of IVF. There are also many clinical cases of polyspermy that are attributable to the penetration of excess sperm after IVF [2,14].

Hundreds of articles have been published on the process of fertilization and polyspermy block in marine animals and mammals including human [2–4]. Two types of mechanisms for polyspermy block have been reported: the “oocyte membrane block” to sperm penetration and the “zona reaction”. The former involves a depolarization of the oocyte membrane caused by the influx of Na^+^, which changes the potential of the oocyte membrane from negative to positive [1]. This change in potential prevents any following sperm from attaching to the oocyte membrane, transiently contributing to polyspermy block. The “oocyte membrane block” or so-called “fast block” to polyspermy occurs in seconds [6–8]; however, it was unlikely to be involved in “fast block” to polyspermy in the fertilization process in mammals [9]. The second proposed mechanism involves a Ca^2+^ oscillation event activated by attachment of the sperm to the oocyte membrane. The subsequent increase in intracellular Ca^2+^ concentration triggers the exocytosis of cortical granules (approximately 1 μm in diameter) from just below the oocyte membrane into the perivitelline space. Enzymes such as hydrolase, proteinase, and peroxidase, which are contained in the cortical granules, prevent the penetration of following sperm by modifying the structure of the sperm receptors such as ZP2 and ZP3 (“slow block” to polyspermy) and by hardening the ZP [5,11]. This “slow block” occurs within approximately 5 to 8 min of oocyte activation and is considered the main mechanism of polyspermy block in humans; however, the mechanistic details of this process remain largely unclear in human [5,11].

The entire fertilization process is extremely delicate and precise, and there is no doubt that multiple layers of safety mechanisms exist to ensure the achievement of normal fertilization. A failure of this safety mechanism is believed to instantly trigger abnormal fertilization, such as polyspermy. However, because it is so difficult to establish laboratory models of the human in vivo fertilization process and few such studies have been conducted, fertilization safety mechanisms operating in human oocytes remain unclear. In addition, dynamic morphological tactics such as TLC have not been applied to analyze the possible mechanisms of polyspermy block.

**Fig. 4:**
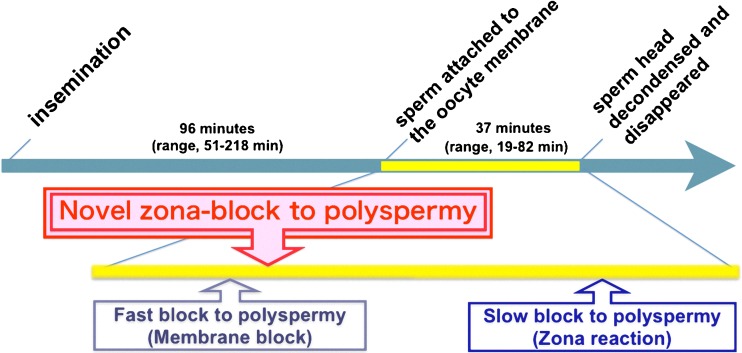
A proposed mechanism for a fast zona-block to polyspermy during the human fertilization process. The findings of our TLC analysis indicate the existence of a novel mechanism of polyspermy block, which takes place in the zona pellucida and differs from the oocyte membrane block to sperm penetration (fast block to polyspermy) and the zona reaction (slow block to polyspermy)

It is true that the TLC analysis presented here still does not exactly represent the in vivo situation. Nevertheless, we believe that these morphological data reveal a novel system for polyspermy block that could occur in human oocytes, and that the images analyzed represent the true dynamic physiology of the human fertilization process. We believe that a novel mechanism of polyspermy block takes place in the ZP, which differs from both the oocyte membrane block to sperm penetration (“fast block” to polyspermy) and the zona reaction (“slow block” to polyspermy) (Fig. 4).

We are planning to elucidate the details of this novel mechanism by further observing the fertilization process of human oocytes using our TLC system.

## Conclusions

By use of our original TLC system, we have succeeded in demonstrating the possible existence of a novel mechanism of polyspermy block in human oocytes. However, the details of this mechanism of polyspermy block are not yet fully understood, and the mechanism we have revealed in this study may be a small part of a highly complicated system. We will continue to analyze the fertilization process of human oocytes by use of the TLC system to reveal the details of this novel mechanism of polyspermy block in human oocytes.
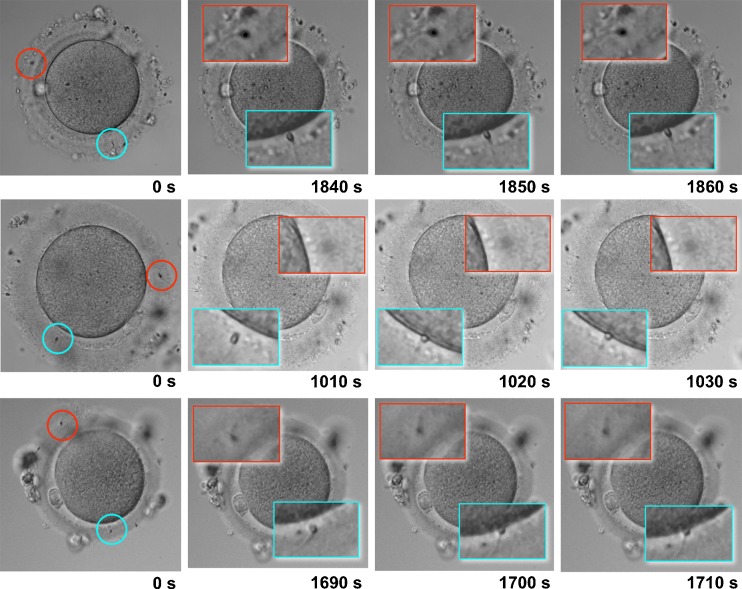
 
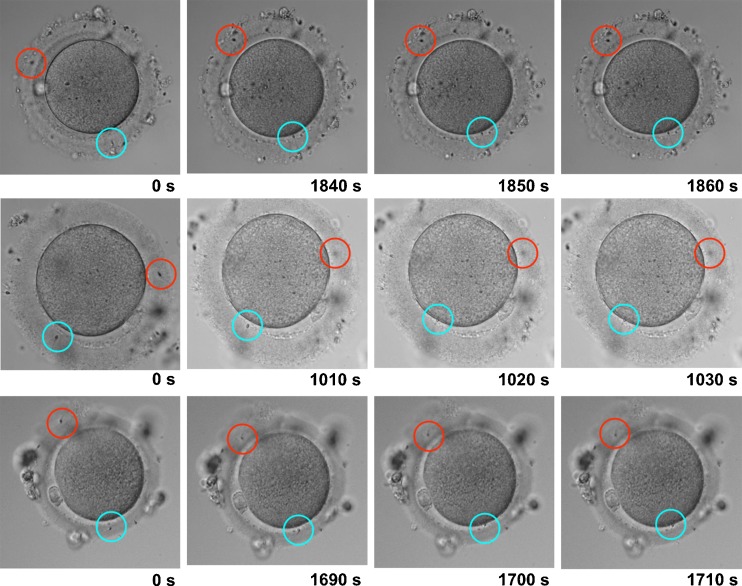
 
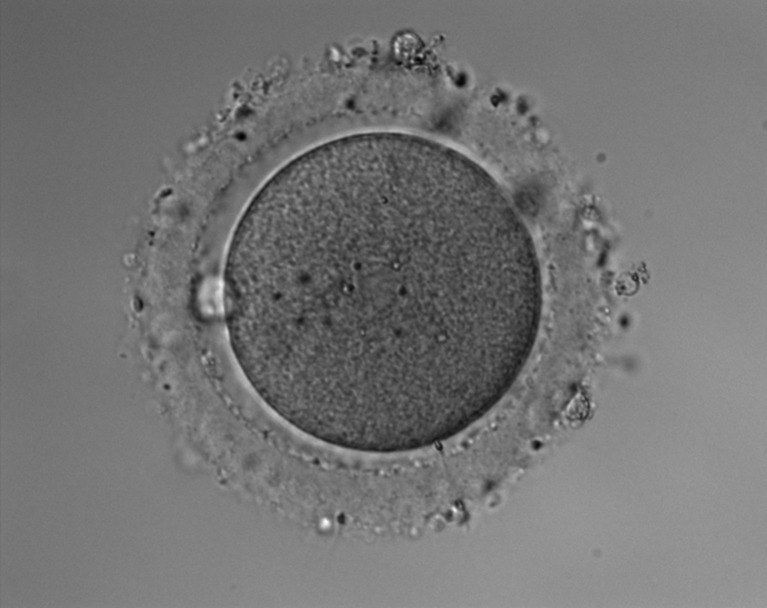


## Electronic supplementary materials

Below is the link to the electronic supplementary material.

Movie 1 This is the first movie to successfully demonstrate the dynamic process of fertilization in human oocytes in vitro [12] whereby once the leading sperm was attached to the oocyte membrane, the following sperm stopped further penetration within the ZP. This phenomenon prompted our hypothesis that a novel mechanism could exist for the polyspermy block in the human fertilization process. (MPG 12897 kb)

Movie 2 This movie represents the same TLC imaging used to make Movie 1. While the leading sperm (blue circle) traveled within the ZP and moved across the perivitelline space, the following sperm (red circle) was also steadily penetrating within the ZP. However, once the leading sperm was attached to the oocyte membrane, the following sperm stopped penetrating within the ZP even though the following sperm was far away from the leading sperm attachment site. (MPG 2680 kb)

Movie 3 The penetration of the following sperm in this case was also inhibited within 10 s after the attachment of the leading sperm to the oocyte. (MPG 2243 kb)

Movie 4 This movie represents the same TLC imaging data used to make Movies 2 and 3. It shows that even though the tail of the following sperm was still moving actively, the sperm did not penetrate further. (MPG 2680 kb)
